# Supplementary material for: Novel ST1926 Nanoparticle Drug Formulation Enhances Drug Therapeutic Efficiency in Colorectal Cancer Xenografted Mice
Source: Nanomaterials (Basel). 2024 Aug 23;14(17):1380. doi: 10.3390/nano14171380 (PMC11396872; doi:10.3390/nano14171380)
Supplement: Supplementary file 1 [file nanomaterials-14-01380-s001.zip › nanomaterials-3069150-supplementary.docx]

­­Type of the Paper (Article)

Novel ST1926 Nanoparticle Drug Formulation Enhances Drug Therapeutic Efficiency in Colorectal Cancer Xenografted mice

Sara Assi ^1^, Berthe Hayar^2^, Claudio Pisano^3^, Nadine Darwiche^2^*, Walid Saad^4^*

^1^ Biomedical Engineering Program, American University of Beirut, Beirut, Lebanon; saa124@mail.aub.edu

^2^ Department of Biochemistry & Molecular Genetics, American University of Beirut, Beirut, Lebanon; bh48@aub.edu.lb (B.H); nd03@aub.edu.lb (N.D)

^3^ Biogem, Institute of Molecular Biology and Genetics, Via Camporeale, 83031 Ariano Irpino (AV), Italy;

claudio.pisano@biogem.it

^4^ Department of Chemical Engineering and Advanced Energy, American University of Beirut, Beirut, Lebanon; ws20@aub.edu.lb

***** Correspondence: nd03@aub.edu.lb (N.D); ws20@aub.edu.lb (W.S)

**HPLC parameters**

**Table S1.** High performance liquid chromatography parameters.

| **Flow rate** | **Wavelength** | **Mobile phase A** | **Mobile phase B** |
| --- | --- | --- | --- |
| 0.5 ml/hr | 320 nm | 0.05% Triethylamine in Water | 0.05% Triethylamine in Methanol |


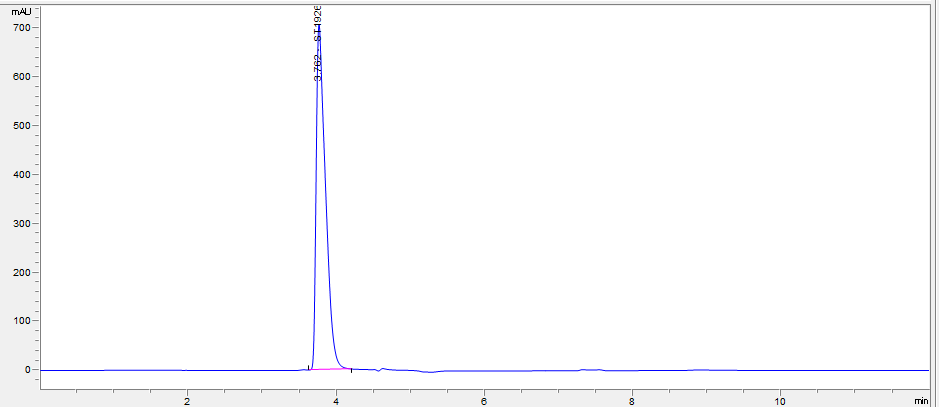

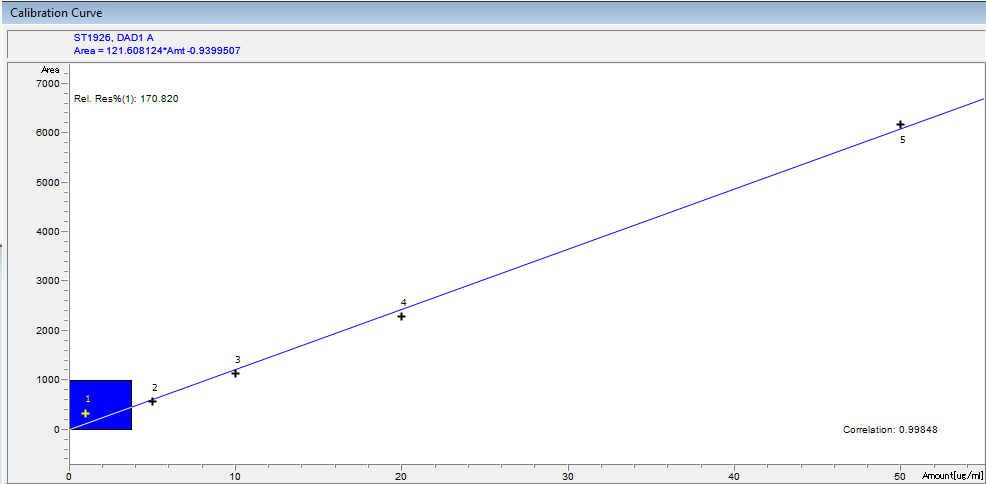


**A**

**B**

**Figure S1.** Analytical Characterization of ST1926 by HPLC **(A)** Chromatogram peak pertaining to ST1926 particles at a concentration of 50 µg/ml; **(B)** Calibration curve for quantification of ST1926 by HPLC: relationship between peak area and concentration, with R^2^=0.998.


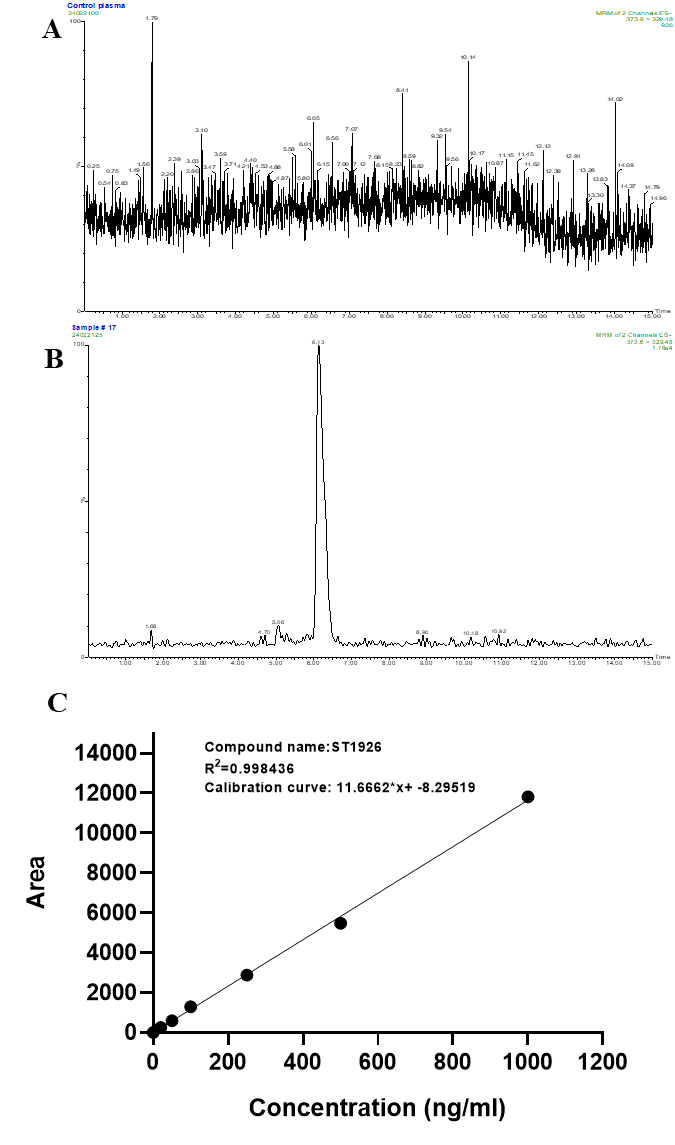


**Figure S2.** Analytical characterization of ST1926 by HPLC-MS/MS. **(A)** Multiple reaction monitoring chromatogram of a mice blank plasma sample; **(B)** Multiple reaction monitoring chromatogram of ST1926 sample (350 ng/ml); **(C)** Calibration curve generated with R^2^=0.998. The calibration curve was built from seven different concentrations of ST1926 spiked with control mice plasma.

**Figure S3**. Calibration curve for quantification of ST1926 by HPLC-QTOF/MS, relationship between peak area and concentration, with R2=0.997.

**Figure S4**. Intensity size and polydispersity index (PDI) of ST1926-nanoparticles. The size and PDI of the polymer-coated ST1926 was determined by dynamic light scattering. Results represent the average of three independent experiments.

**Figure S5**. Size and polydispersity index (PDI) of control-nanoparticles. The size and PDI of the polymer-coated ST1926 was determined by dynamic light scattering initially and after 24 hours. Results represent the average of three independent experiments (± SEM).

**Figure S6.** IC50 curves of ST1926 and ST1926-nanoparticles (NPs) on HCT116 cells post 24 hours treatment. Results are expressed as % of the control group set as 100%. Each data point represents an average of three independent experiments.

**A**

**B**

**Figure S7.** (A) ST1926 and (B) ST1926-nanoparticles (ST1926-NPs) effects on normal-like colorectal cancer cell growth. NCM460 cells were treated with the indicated concentrations of ST1926 or ST1926-NP for up to three days. Cell growth was examined using the MTT colorimetric assay. Results are expressed as percentage of the control group set as 100% and represent the average of two independent experiments ± SD.
